# An investigation of the owner‐ and pet‐related factors that may affect the use of alternative feeding practices in dogs and cats in Hungary

**DOI:** 10.1002/vro2.70004

**Published:** 2024-12-19

**Authors:** Blanka Vékony, Zsanett Bodor, Dániel Sándor Veres, Péter Vajdovich, Erzsébet Mák

**Affiliations:** ^1^ Department of Dietetics and Nutritional Sciences Semmelweis University Faculty of Health Sciences Budapest Hungary; ^2^ Department of Biophysics and Radiation Biology Semmelweis University Budapest Hungary; ^3^ Department of Clinical Pathology and Oncology University of Veterinary Medicine Budapest Hungary

## Abstract

**Background:**

Alternative feeding practices have become increasingly popular among companion animal owners. We sought to identify possible factors behind the choice of alternative feeding types.

**Methods:**

A cross‐sectional study was performed with Hungarian pet owners. Descriptive data analysis and logistic regression models were undertaken to determine factors that may increase the likelihood of adopting alternative feeding practices.

**Results:**

In total, 1007 pet owners completed the questionnaire, of which 789 were dog owners and 218 were cat owners. The type of settlement was identified as a possible factor influencing the choice of application of alternative feeding patterns for dogs and cats. In the case of dogs, the owner's diet variable showed significantly increased odds of choosing an alternative feeding pattern where the owner followed an alternative diet. For cats, the owner's diet did not have a significant effect on choosing alternative feeding practices for these pets.

**Conclusions:**

Owners who followed an alternative diet were more likely to choose an alternative feeding pattern for their dogs but not for their cats. Further study is required to identify additional factors that may influence the owners’ choice of feeding practices and to more widely investigate the feeding habits of cat owners.

## INTRODUCTION

The human‒companion animal interaction could have beneficial or adverse effects on the health and quality of life of pets.^1^, ^2^, ^3^ One of the most important elements in this relationship is the choice and implementation of feeding practices.^4^, ^5^, ^6^ Several factors could influence the choice of feeding practice, including the social determination, knowledge and financial status of the owner.^7^ Additional factors could be the personal eating habits of the owner or the same social and cultural factors that influence people's food choices and consumption^8^, ^9^; additionally, the ‘humanisation’ of companion animals is an added highlight.^10^

In surveys conducted in the early 2000s in the United States and Australia, at least 90% of feeding practices among companion animals were conventional pet food‐based. At that time, there were a significant number of recalls of commercially available pet foods, which are thought to influence owners’ choice of feeding pattern.^11^, ^12^, ^13^ There was a demand from owners to have more influence on the feeding of their pets.^4^, ^14^ This motivation has been accompanied by a growing interest in alternative feeding practices.^10^, ^15^ These may include various alternative/unconventional diets, including vegetarian, vegan,^16^, ^17^ biologically appropriate raw feeding (BARF), raw meat‐based diets (RMBD)^4^, ^6^ and home‐prepared diets.^8^, ^12^, ^13^ If applied without proper knowledge or adequate attention, these alternative diets may result in health concerns such as malnutrition, nutrient deficiencies, imbalances^18^, ^19^, ^20^ or contamination with pathogens.^4^, ^21^, ^22^, ^23^ Considering these possible effects, it is essential to identify factors that may influence owners when they choose alternative feeding patterns.

The aim of this study was to investigate the frequency of choosing alternative diets for pets among Hungarian dog owners and cat owners. The increasing number of pets in Hungary in recent years warrants a study of this topic.^24^ A further aim was to investigate owner‐ and pet‐related factors that increase the likelihood of choosing an alternative feeding pattern. Particularly, alternative diet‐following owners were more likely to choose an alternative feeding pattern for their pets than owners eating a conventional diet.

## METHODS

### Participant recruitment

The participants of the survey had to own at least one pet (dog or cat), be over 18 years old and a Hungarian resident. The respondent had to live with and care for a pet daily. The questionnaire was shared through social media with several pet‐centric groups (including relevant Facebook groups) using the snowball sampling method. According to the ethical statement, before completing the questionnaire, participants were given detailed written information about the purpose of the survey and the expected time needed to complete it. The name and contact details of the person in charge of the survey were indicated in case of further questions. The respondents were informed that their participation was anonymous and voluntary and they could leave the questionnaire at any stage. By answering a certain question at the end of the questionnaire, the respondents were able to give their full consent for the analysis of their unidentified data.

### Data collection and questionnaire

A self‐developed, cross‐sectional questionnaire (Supporting Information S1) was used, and the survey was carried out using Google Forms. The questionnaire was available for 6 months in 2020. The survey contained 29 questions, with an estimated completion time of 15‒20 min. The majority of the questions were closed, with multiple‐choice, Likert scale and free‐text questions.

The first part of the questionnaire focused on general information about the owned pets (age, sex, reproductive status, verbal description and a visual five‐point body condition score [BCS]). The survey included questions about the dietary habits of the owners and the preferred feeding pattern. If the owners fed the pet using an alternative diet, further questions were asked regarding the type of diet, duration, advisor, source of information and usage of treats. Questions related to the socio‐demographic (gender, age, education level and settlement type) and anthropometrical data (height and bodyweight) of the owners.

### Data analysis

#### Descriptive statistical analysis

The owners’ and pets’ socio‐demographical, anthropometrical and characteristics questions were evaluated using descriptive statistics (means, standard deviations and frequencies).

#### Data editing for further analysis

Pet owners were categorised for proper analysis of the data on the basis of a published four‐stage age group definition.^25^ Young adults aged 18‒34 years, middle‐aged owners aged 35‒49 years, late middle‐aged owners aged 50‒65 years and greater than 66 years were placed in the older adults’ category. The respondents’ body mass index (BMI; kg/m^2^) was calculated based on their self‐reported current bodyweight in kilograms and divided by the square of the height in metres (BMI = weight [kg]/height^2^ [m^2^]).

Four categories of weight status were defined according to a published classification.^26^ For a detailed statistical analysis, this was modified into three categories based on low, normal and high BMI values.

Categories were created for educational levels. Respondents with elementary and vocational school certificates formed the ‘basic level’ group. These owners did not have a high‐school certificate according to the Hungarian educational system. The ‘secondary’ group included participants with a high‐school certificate. The third ‘higher’ category included owners with a university degree or higher qualification.

With the owner's diet, those who answered that they did not follow any alternative diet or an omnivorous diet were separated from the other respondents. Those following a therapeutic diet by medical diagnosis were not included in the alternative diet group. The alternative diet group included pet owners who followed a non‐therapeutic diet other than omnivorous, such as vegetarian, vegan, ketogenic, palaeolithic or intermittent fasting.^27^

Owners were requested to provide the pet age in months. For both dogs and cats, life stage assignment was determined based on the American Animal Hospital Association's guidelines.^28^, ^29^ The five‐point BCS system was used for both species according to the AAHA's nutritional assessment guidelines.^30^, ^31^ The visual BCS was asked after the verbal description scale, in the form of a five‐point Likert scale. Dogs and cats were divided into three categories based on the responses received. We classified pets into lower (BCS 1‒2), normal (BCS 3) and higher (BCS 4‒5) groups for both the verbal description and visual BCS.

Responses from owners regarding feeding practices were evaluated individually. Thus, if a therapeutic diet for a diagnosed disease was followed, it was not considered an alternative diet. In addition, all types of feeding other than conventional were classified as part of the alternative group.^8^, ^32^ The ‘raw diet’ subcategory included pets fed a BARF or RMBD, or prey model raw diet. The ‘plant‐based’ subcategory was for pets receiving any type of vegetarian or vegan diet. The ‘free‐from’ subgroup included diets free of some component, such as gluten‐free or grain‐free diets. Diets for suspected future health problems or diseases were classified into the ‘disease prevention’ subgroup. Feeding practices aimed at weight management were classified into a group labelled ‘weight control’, most of them were low‐carbohydrate diets. The ‘other’ subcategories contained pet diets that were mentioned only once or twice with a low frequency, including the human palaeolithic or ketogenic diet.^33^ These categories and subcategories served as variables in the models created in the logistic regression analysis.

#### Inferential statistical models

Two logistic regression models with logit link were used for dogs and cats to identify factors that may have influenced diet selection (alternative diet used vs. not, used as outcome variable). One group of variables was related to the owner (owner's diet, gender and age category) and another group was related to the pet (such as reproductive status).

The final models did not contain interactions and non‐linear terms because we did not detect relevant and significant interactions and non‐linearities (based on common sense, graphs, model fit diagnostics and information criteria). The final models did not contain multicollinear variables. The model's fit and influential points were assessed by diagnostic plots and found to be acceptable. For making inferences, we reported the odds ratios (ORs), likelihood ratios and test *p*‐values for each explanatory variable without multiplicity correction.

Given that the owner's diet may have been (in theory) influenced by the pet's diet, we analysed additional regression models without the owner's diet (to avoid collider bias). To assess the strength of relationship between the pet and the owner's diet, the ORs were determined with a 95% confidence interval (CI) using Fisher's exact test.

All data analyses were performed using Microsoft Excel 2016 and R‐project (R: A Language and Environment for Statistical Computing, R Foundation for Statistical Computing, 2021, v4.1.1; www.R‐project.org/) using the ggplot2 package for descriptive plots (v3.3.5; Ggplot2; https://CRAN.R‐project.org/package=ggplot2), the rms package (v6.2.0; https://CRAN.R‐project.org/package=rms) for assessing non‐linearity in regression models and the DHARMa package (v0.4.4; DHARMa: Residual Diagnostics for Hierarchical [Multi‐Level/Mixed] Regression Models; http://florianhartig.github.io/DHARMa/) for model diagnostic methods.

## RESULTS

### Dog and cat owners—socio‐demographic results

In total, 1007 pet owners completed the questionnaire: 789 (78.35%) owned dogs and 218 (21.65%) owned cats. The detailed results of pet owners (*n* = 1007) distribution of age categories, level of education, type of settlement and weight status are given in Table S1.

### Characteristics of owned dogs and cats

The sex distribution of pets was almost identical: 50.8% male and 49.2% female. Further detailed results (sex, age category, reproductive status and visual BCS) of owned pets (*n* = 1007) are given in Table S2.

### Feeding practices for owned dogs and cats

**FIGURE 1:**
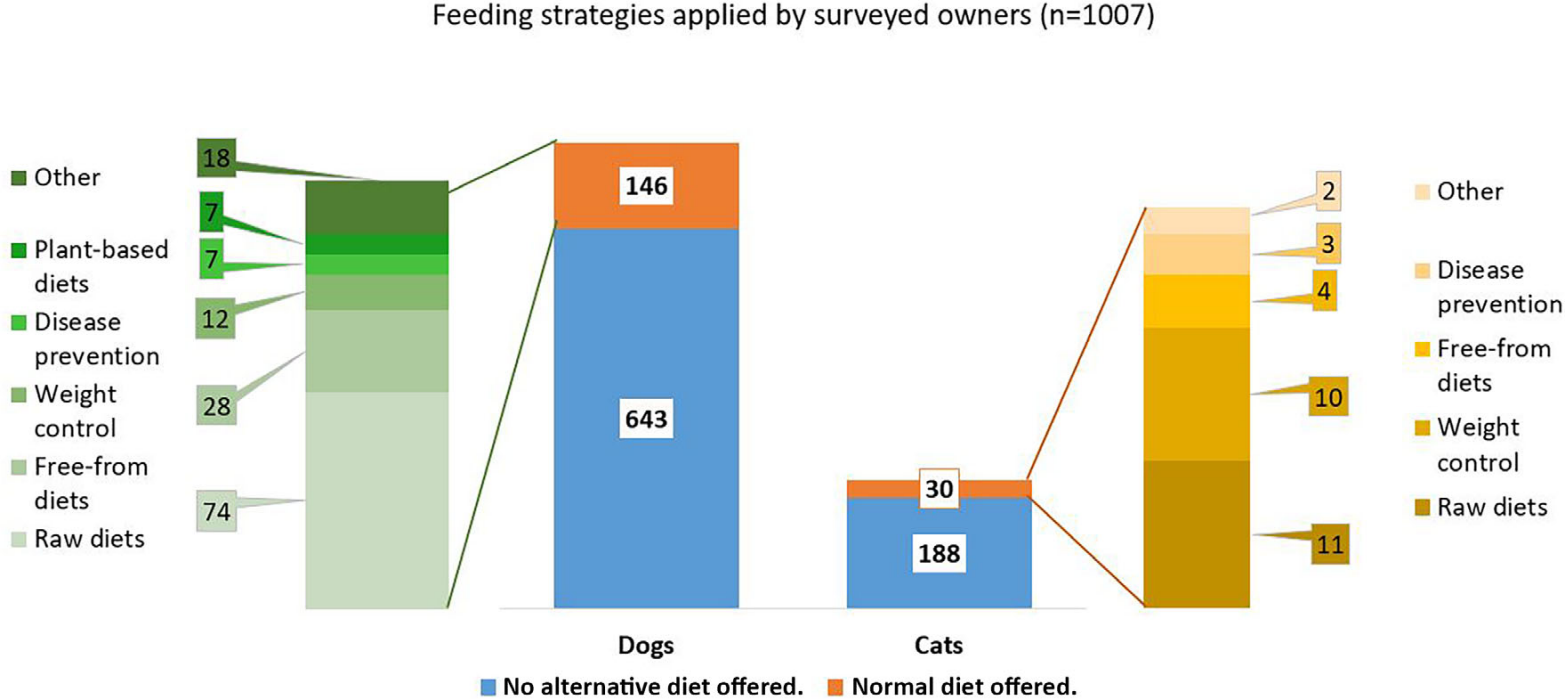
Distribution of various pet feeding practices reported by owners (*n* = 1007). Numbers represent the number of pets per group.

Descriptive analysis of the pets related to the feeding practices (Figure 1) showed that, in total, 831 (82.5%) participants did not offer any alternative/unconventional diet to their pet. A total of 146 (18.5%) dog owners and 30 (13.8%) cat owners offered some kind of alternative diet. The most common feeding practice for each species was the raw diet, with 74 (50.7%) dogs and 11 (36.7%) cats. The second most popular diet was the ‘free from’ category for 28 (19.2%) dogs and ‘weight control’ for 10 (33.3%) cats. The third favourite group of 12 (8.2%) dogs was ‘weight control’ and the ‘free from’ category for four (13.3%) cats. No cat owner answered that they preferred a plant‐based diet for their cat, whereas seven (4.9%) dog owners answered that they selected some form of plant‐based diet. The ‘others’ category included palaeolithic, ketogenic and ‘self‐developed’ diets.

### Dietary choices of pet owners

We examined and categorised the types of diets followed by pet owners. The participants were divided into two groups according to whether they followed an alternative diet or not. A descriptive analysis of companion animal owners showed that 809 (80.3%) did not follow an alternative diet. In contrast, a total of 147 (18.6%) dog owners and 51 (23.4%) cat owners followed any alternative diet. The most popular subcategory was any type of weight management practices (*n* = 85; 43.0% of alternative diet followers). This was followed by a group of plant‐based dieters (*n* = 68; 34.3% of alternative diet followers). In addition, pet owners also responded to the palaeolithic (*n* = 18; 9.1%), ketogenic (*n* = 10; 5.1%), free‐from (*n* = 9; 4.5%), dissociated (*n* = 5; 2.5%) and intermittent fasting (*n* = 3; 1.5%) diets. The proportions of owners who followed an alternative diet and then chose an alternative diet for their pets are summarised in Table 1.

**TABLE 1:** Main diet/feeding type comparison of owners and their pet (dog/cat).

	Alternative diet offered to their cat or dog		
Owner following an alternative diet	Yes (*n* = 176)	No (*n* = 831)	Total (*n* = 1007)
Dog owners (*n* = 789)			
Yes	46 (31.5%)	101 (15.7%)	147 (18.6%)
No	100 (68.5%)	542 (84.3%)	642 (81.4%)
Cat owners (*n* = 218)			
Yes	9 (30.0%)	42 (22.3%)	51 (23.4%)
No	21 (70.0%)	146 (77.7%)	167 (76.6%)

*Note*: The percentages are in proportion to the total × number of dog and cat owners.

In total, there were seven cases where the diet of the owner and the feeding pattern of the pet were the same. In four of these cases, both the owner and their dog were consuming some type of weight control diet. The same conditions occurred twice among cat owners and their cats. In one case, both the owner and the pet were receiving a free‐from diet. Six of the owners following a vegetarian diet chose a raw diet for their dog.

### Statistical analysis of influencing factors

According to the logistic regression analysis, we found a statistically significant effect in the case of two factors related to the owners. The first was the owner's diet (*p* < 0.001), where the sub‐variable alternative diet (OR = 2.78; 95% CI 1.80‒4.28) showed significantly increased odds than the conventional diet. That is, owners following an alternative diet were more likely (2.78 times higher odds) to choose an alternative type of diet for their dog.

From the kind of settlement (*p* = 0.017), the town had the highest odds (OR = 2.16; 95% CI 1.26‒3.86) versus the village for alternative feeding application. At the same time, capital resulted in an increased OR (1.98; 95% CI 1.09‒3.70) versus village. It was found that dogs living in the capital or urban areas had a significantly higher probability of being fed an alternative diet than those living in rural areas.

The gender of owners had no significant effect on the likelihood of choosing an alternative diet for dogs. Although the estimated odds were higher for female owners (OR = 1.65; 95% CI 0.89‒3.31), whereas the reference variable was the male owners.

The calculation of the probability of adopting an alternative feeding pattern for dogs in relation to neutering did not have a significant result in relation to the visual BCS. The odds for the age category and verbal description BCS of dogs were not statistically significant. Logistic regression did not result in significant odds when examining factors from the owners’ side, including age category, level of education and weight status. The results of the logistic regression model for dogs are presented in Table 2.

**TABLE 2:** Analysis of factors that may have influenced the choice of alternative feeding practices for dogs.

Variables	*p*‐Value	Odds ratio	95% confidence interval
**Related to dogs**			
Sex	0.5395		
Male		Reference	
Female		1.13	0.77‒1.67
Age category	0.395		
Minor		Reference	
Young adult		1.97	0.71‒7.02
Mature adult		2.12	0.77‒7.52
Senior		1.61	0.56‒5.82
Reproductive status	0.092		
Intact		Reference	
Neutered		1.44	0.94‒2.21
Verbal description (Body Condition Score, BCS)	0.304		
Normal		Reference	
Lower		0.75	0.28‒1.94
Higher		2.54	0.70‒10.54
Visual BCS	0.036		
Normal		Reference	
Lower		2.67	1.26‒5.49
Higher		0.83	0.20‒2.95
**Related to owners**			
Owner's diet	0.0001		
No alternative		Reference	
Alternative		2.78	1.80‒4.28
Gender	0.119		
Male		Reference	
Female		1.65	0.89‒3.31
Age category	0.814		
Late middle‐aged, senior		Reference	
Young adult		0.98	0.56‒1.72
Middle‐aged adult		0.87	0.50‒1.51
Type of settlement	0.017		
Village (rural)		Reference	
Town (urban)		2.16	1.26‒3.86
Capital		1.98	1.09‒3.70
Level of education	0.792		
Basic level		Reference	
Secondary		1.30	0.60‒3.10
Higher		1.19	0.56‒2.81
Weight status (body mass index) category	0.5505		
Normal		Reference	
Lower		1.28	0.56‒2.75
Higher		0.85	0.56‒1.28

Logistic regression was performed to examine the odds of alternative feeding pattern choices in the light of each variable among cats. The analysis showed a significant effect of the type of settlement (*p* = 0.027) and verbal description BCS (*p* = 0.031) factors. In the case of type of settlement, there was a lower OR for both sub‐variables, capital (OR = 0.69; 95% CI 0.22‒2.23) and town (OR = 0.24; 95% CI 0.07‒0.81) compared to rural area (village). However, if CIs were taken into consideration in the case of verbal description BCS (cats with higher BCS; OR = 12.24; 95% CI 1.26‒147.35 and cats with lower BCS; OR = 7.67; 95% CI 0.66‒217.86) the result did not show a clear effect.

In contrast to dogs, the examined owner's diet variable did not give a significant result in the case of cats. Although the point estimate (OR = 2.11) of the alternative diet sub‐variable was increased among cats this could not be evaluated as a significant odds increase because of the resulting *p*‐value and wide CI (95% CI 0.78‒5.54). No significant effects were found in the probability of the application of alternative diets for other cat‐related variables including neutering, visual BCS, sex and age category. Analysis of owner‐related variables, including age group, gender, level of education and BMI value did not result in significant ORs for feeding type selection. Although significant odds were not confirmed for the majority of variables, assessing some sub‐variables resulted in an elevated OR compared to the reference group. The detailed results of the logistic regression model for cats are shown in Table 3.

**TABLE 3:** Analysis of factors that may have influenced the choice of alternative feeding practices for cats.

Variables	*p*‐Value	Odds ratio	95% confidence interval
**Related to cats**			
Sex	0.796		
Male		Reference	
Female		0.89	0.38‒2.14
Age category	0.114		
Minor		Reference	
Young adult		4.17	0.98‒31.24
Mature adult		1.97	0.30‒17.50
Senior		1.37	0.21‒12.16
Reproductive status	0.056		
Intact		Reference	
Neutered		6.13	0.96‒128.88
Verbal description BCS	0.031		
Normal		Reference	
Lower		7.67	0.66‒217.86
Higher		12.24	1.26‒147.35
Visual BCS	0.0822		
Normal		Reference	
Lower		0.25	0.01‒2.22
Higher		0.11	0.01‒1.04
**Related to the owners**			
Owner's diet	0.139		
No alternative		Reference	
Alternative		2.11	0.78‒5.54
Gender	0.381		
Male		Reference	
Female		1.84	0.50‒9.26
Age category	0.484		
Late middle‐aged, senior		Reference	
Young adult		1.17	0.31‒5.13
Middle‐aged adult		1.87	0.55‒7.71
Type of settlement	0.027		
Village (rural)		Reference	
Town (urban)		0.24	0.07‒0.81
Capital		0.69	0.22‒2.23
Level of education	0.83		
Basic level		Reference	
Secondary		0.85	0.09‒19.18
Higher		1.13	0.13‒24.89
Body mass index value	0.5967		

The additional analysis, where the regression models did not contain the owner's diet variable, showed similar OR and *p*‐values for the explanatory variable. The Fisher's test showed similar OR and *p*‐values regarding the relation between the pet and owner diets.

## DISCUSSION

According to our findings out of the 1007 pets surveyed, 176 (17.5%) received an alternative feeding pattern, including 18.5% for dogs and 13.8% for cats. Probably raw feeding has the most rapidly growing number of followers and our results reflect this tendency. In the alternative/unconventional diets, the category ‘raw diets’ was the most common reported (48.3%; 50.7% of dogs and 36.7% of cats). One survey reported on raw versus non‐raw diets for 3212 dogs, with 1754 (54.6%) receiving a raw meat diet (RMD).^6^ Another study reported that 20% of the pets examined were fed a diet containing raw animal products (RAPs).^34^ A subsequent study reported that 66% of dogs and 53% of cats received a RAP diet.^10^

The prevalence of providing plant‐based diets was 0.9% for all dogs and 4.8% for alternative feeding patterns adopted in dogs. A study of English‐speaking pet owners reported that 1.6% of respondents fed their pets a plant‐based diet, whereas 45% were interested in this feeding style.^17^ Although there is a difference with our study, it is important to highlight that plant‐based feeding patterns are being adopted by Hungarian dog owners. It is considered an interesting result that overall, the second most frequently nominated alternative feeding category was the ‘free‐from diets’ (*n* = 32; 18.2% of alternative feeding patterns). These feeding practices were used by owners without any veterinary prescription or diagnosis. The decision by owners to adopt these feeding patterns is probably motivated by certain marketing strategies rather than scientific evidence showing any health advantage for dogs and cats.^35^, ^36^ Another possible reason could be a misinterpretation of the labelling of pet food, which could refer to ingredients or even calorie content.^36^

In the statistical analysis, we investigated pet‐ and owner‐related factors from the point of view of alternative feeding pattern selection. In the case of dogs, no significant results were obtained for any of the factors related to the dogs. One study reported that the variables for pet dogs of sex, breed and age were significantly associated with the choice of a RMD among UK dog owners.^6^ According to our results for cats, the verbal description BCS factor showed a significant result. However, considering the CIs obtained, the result did not show a clear effect. With cats and diets, variables that have been studied in more depth include the development of overweight and obesity.^37^, ^38^

The analysis of owner‐related factors showed that the owner's diet might be associated with what diet dogs were offered; however, in the case of both species, there was an association between type of settlement. One study reported that Australian dog owners living in the city preferred to choose non‐commercial food.^39^ In contrast, dog owners living outside a city environment were more likely to use an alternative feeding pattern in one study.^40^ Similar observations were reported among pet owners in New Zealand living in rural, non‐urban areas, who were more likely to choose an RMBD for their pets.^41^ The majority of Hungarian people who are alternative dieters are reported to live in a city (88.9%).^42^ These diverse results may suggest that dog owners in the Hungarian countryside are less likely to be involved in following an alternative diet and adopting an alternative feeding type than those living in a city or town. However, the results among Hungarian cat owners were similar to the results of studies of dog owners. Based on these results, we hypothesise that the type of settlement should be considered as a possible factor influencing the choice of feeding practices. Clarification, it would be important to investigate the income or perhaps even marital status as a factor related to the owners.

Limitations of the study included the method used for data collection and the self‐developed questionnaire. The sample used in the survey might not be representative of all pets and owners in Hungary. The results for cat owners and cats should have considered the limited number of samples. We did not investigate the level of the human‒animal bond and its possible influence on the choice of feeding practices. Additionally, socio‐demographic factors beyond gender, age, level of education and type of settlement were not considered.

In conclusion, we consider that factors that increase the probability of choosing alternative feeding practices could be identifiable. Further investigation is required to identify these factors in more depth (related to owners and pets) and the motivations behind the process. These could determine future intervention points—where professionals can get involved in educating owners who adopt alternative feeding patterns for their pets.

## AUTHOR CONTRIBUTIONS

Blanka Vékony and Erzsébet Mák were responsible for the study design and data collection. Blanka Vékony, Dániel Sándor Veres and Zsanett Bodor conducted the data analysis. Blanka Vékony and Zsanett Bodor interpreted the data. Blanka Vékony wrote the manuscript. Zsanett Bodor and Péter Vajdovich reviewed the final manuscript.

## CONFLICTS OF INTEREST

The authors declare they have no conflicts of interest.

## FUNDING INFORMATION

The authors recieved no specific funding for this work.

## ETHICS STATEMENT

The authors acknowledged the ethical policies of the journal, as noted on the journal's author guidelines page. The study was fully approved by the Semmelweis University Regional and Institutional Committee of Science and Research Ethics (approval no. 173/2020). Study participants provided full consent for their unidentified data to be analysed for research purposes.

## Supporting information



Supporting information

## Data Availability

The data supporting this study's findings are available on request from the corresponding author.
