# Supplementary material for: An investigation of the owner‐ and pet‐related factors that may affect the use of alternative feeding practices in dogs and cats in Hungary
Source: Vet Rec Open. 2024 Dec 19;11(2):e70004. doi: 10.1002/vro2.70004 (PMC11656396; doi:10.1002/vro2.70004)
Supplement: Supplementary file 1 — Supporting information [file VRO2-11-e70004-s001.pdf]

# Supporting Information

## S1 Self-developed, cross-sectional questionnaire

Effects of modern-day pet keeping on current day society

Dear Participant!

By participating in this study you can help my research about the effects of pet keeping onlifestyle and nutrition. Your participation is completely voluntary and anonymous. The questionnaire can be cancelled at any stage of the process. To fill this questionnaire, it takes about 15-20 minutes. Data provided by you will only be used for this experiment and will not be given to any third party person or company!

Guide for completing the questionnaire:

- If you had more than one pet, please only complete this questionnaire for one of them.
- Before answering please read carefully all possible answers.
- Please always choose the best possible answer.
- In some cases, multiple answers are allowed.
- Questions marked with * are obligatory.
- In case of free text answers try to give short answer!

Thank you for your participation!
(The ethical research approval number: 173/2020; contact the head of research: Blanka Vékony, se.vekonyblanka@gmail.com)

Questions about your pet I.

1. Do you own a cat or a dog? *
You can choose only one option!

- ◯ Dog
- ◯ Cat *Jump to question no.: 3*

Question only for dogs

2. Into what size range dose your dog belongs to? *
You can choose only one option!

- ◯ "Mini" dog (max. 3kg)
- ◯ Short statured (between 4,5-11,5 kg - withers: between 23-38 cm)
- ◯ Mid statured (between 11,5-30 kg - withers: between 38-68,5 cm)
- ◯ Big statured (between 30-45 kg - withers: between 53,5-70 cm)
- ◯ Large statured (equals to or more than 50 kg - withers: between 68,5-86,5 cm)

Questions about your pet II.

3. Age of your pet? *
Please, give your answer in the following format: ......year or ............................ month!

_______________________________

4. Gender of your pet? *

You can choose only one option!

- Male
- Female

5. Is your pet after neutering? *

*You can choose only one answer!*

- Yes
- No

Questions about your feeding habits I.

6. Do you consider your pet's weight either: *

1 -thin/lean, 2 -moderately thin/lean, 3 -normal, 4 -overweight, 5 -obese

*You can choose only one answer!*

| | 1 | 2 | 3 | 4 | 5 | |
|---|---|---|---|---|---|---|
| Lean | | | | | | Obese |

7. Which picture matches to your pet the best? *

*You can choose only one answer!*

- 1st picture
- 2nd picture
- 3rt picture
- 4th picture
- 5th picture

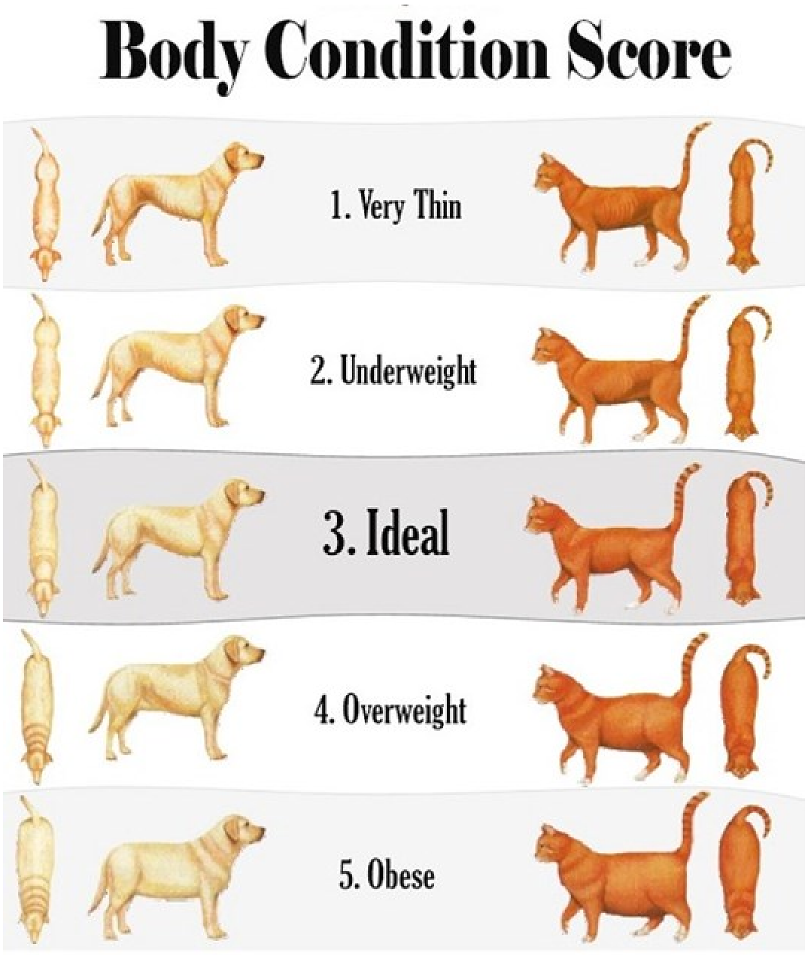

8. Do you feed your pet according to any specific diet or alternative diet? *
*You can choose only one answer!*

- Yes
- No *Jump to question no.: 16*

Questions about your pet's diet I.

9. Do you apply this diet because of diagnosed illness(es)? *
*You can choose only one answer!*

- Yes
- No

10. In this case which diet do you apply? *
*You can choose only one answer!*

- Ketogenic diet
- Paleolithic diet (paleo diet)
- B.A.R.F.
- Vegan diet
- Vegetarian diet
- Food allergy
- Liver-care diet
- Kidney-care diet
- Else:

11. Since what time do you apply this diet? *
Please, give your answer in the following format: ......year or ............................ month!

12. Do you give treats to your pet? *
*You can choose only one answer!*

- Yes
- No

13. Are these treats fit into the diet? *
*You can choose only one answer!*

- Yes
- No

14. Who suggested this diet for your pet? *
You can choose multiple options!

- [ ] Vet
- [ ] Pet trainer
- [ ] Breeder
- [ ] Pet nutritionist

- [ ] Else:

15. Since you have started to apply this diet to your pet what are the main sources of information about feeding? *
You can choose multiple options!

- [ ] Internet
- [ ] Newspaper
- [ ] Own experience
- [ ] Literature, professional books
- [ ] Vet
- [ ] Breeder
- [ ] Pet trainer
- [ ] Facebook groups
- [ ] Else:

Demographic data

Information regarding you (Owner).

16. Your age (in years)? *

17. Your gender? *

*You can choose only one answer!*

- Female
- Male

18. What sized city do you live in? *

*You can choose only one answer!*

- Capital
- County seat
- City
- Small city
- Village or smaller

19. Highest level of education? *
*You can choose only one answer!*

- Less than primary school
- Primary school

Tradesman certificate / Vocational school

High school / Secondary school

BSc. degree

MSc. degree

Ph.D. degree

20. Your height? *
Please, give your answer in centimetre (cm)!

21. Your weight? *
Please, give your answer in kilogram (kg)!

22. Do you consider yourself to be either: *
1 -Thin/lean, 2 -moderately thin/lean, 3 -normal, 4 -overweight, 5 -obese
*You can choose only one answer!*

| | 1 | 2 | 3 | 4 | 5 | |
|---|---|---|---|---|---|---|
| Lean | | | | | | Obese |

Questions about your eating habits I.

23. Do you eat according to any specific diet or fashion diet? *

*You can choose only one answer!*

Yes

No

24. Do you apply this diet due to diagnosed illness(es)?

*You can choose only one answer!*

Yes

No

25. Since what time do you apply this diet? *
Please, give your answer in the following format: ......year or ............................ month! If you have answered NO for the previous question, please write 0 (zero) as an answer!

26. Which diet type do you apply? *

*You can choose only one answer!*

Paleolithic diet (paleo diet)

Vegan diet

Any of the vegetarian diets

Food allergy or intolerance

Raw food diet

Ketogenic diet

Low carb diet

Intermittent fasting

I don't follow any alternative/ fad diet

Else:

Control question

27. Do you feed your pet according to any specific diet or alternative diet? *

*You can choose only one answer!*

Yes

No

Thank you for your time and honest answers!

28. If you have any comment, or remark about the questionnaire you can write that here!

29. Do you consent to your answers being used in the research?

Yes

No

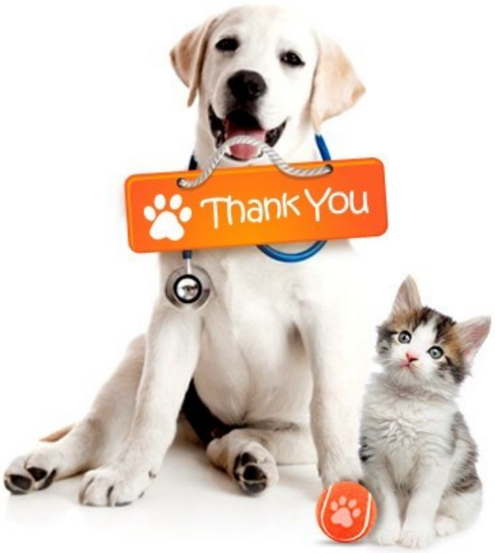

## Table S1 Socio-demographic results for the dog and cat owners

| | Dog owners | Cat owners | Total |
|---|---|---|---|
| **Gender** | | | |
| Female | 686 (86.9%) | 191 (87.6%) | 877 (87.1%) |
| Male | 103 (13.1%) | 27 (12.4%) | 130 (12.9%) |
| **Age categories (years)** | | | |
| young adult (18-34) | 336 (42.6%) | 94 (43.1%) | 430 (42.7%) |
| middle-aged adult (35-49) | 311 (39.4%) | 92(42.2%) | 403 (40.0%) |
| late middle-aged adult (50-65) | 128 (16.2%) | 28 (12.9%) | 156 (15.5%) |
| older adults (66≤) | 14 (1.8%) | 4 (1.8%) | 18 (1.8%) |
| **Level of education** | | | |
| Elementary | 22 (2.8%) | 2 (0.9%) | 24 (2.4%) |
| Vocational school certificate | 39 (4.9%) | 8 (3.7%) | 47 (4.7%) |
| High school certificate | 301 (38.1%) | 76 (34.9%) | 377 (37.4%) |
| Bachelor's degree | 255 (32.3%) | 77 (35.3%) | 332 (33.0%) |
| Master's degree | 157 (19.9%) | 45 (20.6%) | 202 (20.1%) |
| PhD | 15 (1.9%) | 10 (4.6%) | 25 (2.5%) |
| **Type of settlement** | | | |
| capital | 238 (30.2%) | 76 (34.9%) | 314 (31.2%) |
| town | 379 (48.0%) | 106 (48.6%) | 485 (48.2%) |
| village | 172 (21.8%) | 36 (16.5%) | 208 (20.7%) |
| **Weight status (Body Mass Index)** | | | |
| Underweight (>18.5 kg/m$^{2)}$ | 46 (5.8%) | 13 (6.0%) | 59 (5.9%) |
| Normal weight (18.5-24.99 kg/m$^2$) | 418 (53.0%) | 109 (50.0%) | 527 (52.3%) |
| Overweight (≥25.0 kg/m$^2$) | 210 (26.6%) | 56 (25.7%) | 266 (26.4%) |
| Obese (≥30.0 kg/m$^2$) | 115 (14.6%) | 40 (18.3%) | 155 (15.4%) |
| **Total (person)*** | **789** | **218** | **1007** |

Percentages are in proportion to the total* number of dog/cat/total owners

## Table S2 Characteristics of dogs and cats according to sex, age, reproductive status and five-point Body condition score (BCS)

| | Dogs | Cats | Total |
|---|---|---|---|
| **Sex** | | | |
| Female | 398 (50.4%) | 97 (44.5%) | 495 (49.2%) |
| Male | 391 (49.6%) | 121 (55.5%) | 512 (50.8%) |
| **Age category** | | | |
| puppy / kitten | 48 (6.1%) | 30 | 78 |
| young adult | 232 | 121 | 353 |
| mature adult | 289 | 28 | 317 |
| senior | 220 | 39 | 259 |
| **Reproductive status** | | | |
| Intact | 465 (58.9%) | 193 (88.5%) | 658 (65.3%) |
| Neutered | 324 (41.1%) | 25 (11.5%) | 349 (34.7%) |
| **Five-point Body Condition Score** | | | |
| 1 - very thin | 13 (1.7%) | 1 (0.5%) | 14 (1.4%) |
| 2 - moderately thin | 67 (8.5%) | 26 (11.9%) | 93 (9.2%) |
| 3 - normal | 614 (77.8%) | 141 (64.7%) | 755 (75.0%) |
| 4 - overweight | 86 (10.9%) | 44 (20.2%) | 130 (12.9%) |
| 5 - obese | 9 (1.1%) | 6 (2.7%) | 15 (1.5%) |
| **Total (pet*)** | **789** | **218** | **1007** |

Percentages are in proportion to the total* number of dog/cat
